# Protection from Inflammatory Organ Damage in a Murine Model of Hemophagocytic Lymphohistiocytosis Using Treatment with IL-18 Binding Protein

**DOI:** 10.3389/fimmu.2012.00239

**Published:** 2012-08-08

**Authors:** Laura Chiossone, Sandra Audonnet, Bruno Chetaille, Lionel Chasson, Catherine Farnarier, Yael Berda-Haddad, Stefan Jordan, Ulrich H. Koszinowski, Marc Dalod, Karin Mazodier, Daniela Novick, Charles A. Dinarello, Eric Vivier, Gilles Kaplanski

**Affiliations:** ^1^Centre d’Immunologie de Marseille-Luminy, INSERM, U 1104Marseille, France; ^2^CNRS, UMR7280Marseille, France; ^3^Aix Marseille Université, UM2Marseille, France; ^4^Laboratoire d’Immunologie, Assistance Publique – Hôpitaux de Marseille, Hôpital de la ConceptionMarseille, France; ^5^Laboratoire d’Anatomopathologie, Institut Paoli-CalmettesMarseille, France; ^6^Laboratoire d’Hématologie, Assistance Publique – Hôpitaux de Marseille, Hôpital de la ConceptionMarseille, France; ^7^Max von Pettenkofer InstitutMünich, Germany; ^8^Service de Médecine Interne, Assistance Publique – Hôpitaux de Marseille, Hôpital de la ConceptionMarseille, France; ^9^Department of Molecular Genetics, The Weizmann Institute of ScienceRehovot, Israel; ^10^Division of Infectious Diseases, University of ColoradAurora, CO, USA

**Keywords:** IL-18, IL-18 binding protein, natural killer cells, hemophagocytic lymphohistiocytosis, macrophage activation syndrome

## Abstract

Hemophagocytic lymphohistiocytosis (HLH) is a life-threatening condition due to the association of an infectious agent with lymphocyte cytotoxicity defects, either of congenital genetic origin in children or presumably acquired in adults. In HLH patients, an excess of lymphocyte or macrophage cytokines, such as IFN-γ and TNFα is present in serum. In animal models of the disease, IFN-γ and TNF-α have been shown to play a central pathogenic role. In humans, unusually high concentrations of IL-18, an inducer of IFN-γ, and TNF-α have been reported, and are associated with an imbalance between IL-18 and its natural inhibitor IL-18 binding protein (IL-18BP) resulting in an excess of free IL-18. Here we studied whether IL-18BP could reduce disease severity in an animal model of HLH. Mouse cytomegalovirus infection in perforin-1 knock-out mice induced a lethal condition similar to human HLH characterized by cytopenia with marked inflammatory lesions in the liver and spleen as well as the presence of hemophagocytosis in bone marrow. IL-18BP treatment decreased hemophagocytosis and reversed liver as well as spleen damage. IL-18BP treatment also reduced both IFN-γ and TNF-α production by CD8^+^ T and NK cells, as well as Fas ligand expression on NK cell surface. These data suggest that IL-18BP is beneficial in an animal model of HLH and in combination with anti-infectious therapy may be a promising strategy to treat HLH patients.

## Introduction

Hemophagocytic lymphohistiocytosis (HLH) is a life-threatening syndrome affecting children and adults, characterized by persistent fever, hepato-splenomegaly, cytopenia, hypertriglyceridemia, hyperferritinemia, and the presence of hemophagocytosis in bone marrow or other tissues (Henter et al., 2007; Janka, 2007). The disease can be of genetic origin, affecting children before 6 months of age, secondary to various mutations affecting genes coding for proteins involved in lymphocyte cytotoxicity, the prototypic form being perforin deficiency in familial HLH (F-HLH) type 2 (Stepp et al., 1999; Henter et al., 2007; Janka, 2007). Other mutations have been reported to be associated with F-HLH, such as mutations affecting Munc 13-4 in F-HLH type 3, syntaxin 11 in F-HLH type 4, Munc 18-2 in F-HLH type 5, as well as mutations of Rab27a in Griscelli syndrome and Lyst in Chediak–Higashi syndrome (Pachlopnik Schmid et al., 2010). In this genetic context of severely impaired lymphocyte cytotoxicity, infection often appears to be a necessary trigger to induce the disease (Henter et al., 2007; Pachlopnik Schmid et al., 2010).

In adults, HLH is encountered in association with a variety of underlying conditions notably viral infections due to herpes virus family members such as Epstein–Barr virus (EBV), herpes simplex, and cytomegalovirus (CMV; Henter et al., 2007; Rouphael et al., 2007). Other intracellular infections, hematological malignancies notably T cell lymphoma but also auto-immune diseases such as systemic lupus erythematosus and particularly inflammatory diseases such as systemic onset juvenile idiopathic arthritis (also known as Still’s disease) are also associated with secondary HLH (S-HLH; Ravelli, 2002; Lambotte et al., 2006). In adult patients, lymphocyte cytotoxicity is also frequently compromised due to previous immunosuppressive treatments or to unknown mechanisms (Grom, 2004; Mazodier et al., 2005). In addition, some adult patients affected by S-HLH carry mutations of genes involved in F-HLH, such as perforin and Munc 13-4, although in less severe forms than those observed in F-HLH (Clementi et al., 2002; Zhang et al., 2008). These observations have led to the concept that the combination of an infectious trigger with severely impaired lymphocyte cytotoxicity is required to induce HLH in patients (Pachlopnik Schmid et al., 2010). Another characteristic of this disease is the large excess of cytokines secreted by lymphocytes (IFN-γ, sIL-2R) and macrophages (TNF-α, GM-CSF) which is commonly observed in these patients and may be responsible for most of the clinical and biological symptoms (Henter et al., 1991).

Progress in the pathogenesis of HLH has been obtained in animal models. Several models have been reported to date, they each combine mutations affecting lymphocyte cytotoxicity plus a viral infection (Kägi et al., 1994; Walsh et al., 1994; Jordan et al., 2004; Van Dommelen et al., 2006; Crozat et al., 2007; Pachlopnik Schmid et al., 2008). The more suitable model appears to be lymphocytic choriomeningitis (LCMV) infection in perforin-1 knock-out mice (Perf1 KO; Kägi et al., 1994; Walsh et al., 1994; Jordan et al., 2004). In this model, IFN-γ but not other cytokine blockade, can increase survival therefore identifying IFN-γ as a major cytokine in HLH pathogenesis (Jordan et al., 2004). While LCMV is a non-cytopathic virus (Kägi et al., 1994), HLH in humans is commonly associated with severe sepsis or cytopathic viral infections, such as CMV, fatal H5N1, or H1N1 influenza A viruses (Janka et al., 1998; Yuen et al., 1998; Riedemann et al., 2003; Rouphael et al., 2007). Another murine model has been described consisting in mouse CMV (MCMV) infection in Perf1 KO mice (Van Dommelen et al., 2006). In that model, both IFN-γ and TNF-α appear to be required for tissue damage (Van Dommelen et al., 2006).

IL-18 is a member of the IL-1 family produced by macrophages, peripheral blood mononuclear cells (PBMCs), or dendritic cells as a 24 kDa precursor inactive form which is cleaved by caspase-1 in a 18 kDa mature biologically active molecule (Dinarello and Kaplanski, 2005; Dinarello, 2007). IL-18 induces IFN-γ production by NK cells and T cells in combination with IL-12 (Dinarello, 2007). IL-18 is also able to induce TNF-α and chemokine secretion by macrophages (Dinarello, 2007). IL-18 binding protein (IL-18BP) is the natural inhibitor of IL-18 *in vivo*, and is a potent inhibitor of IFN-γ production (Novick et al., 1999). Various studies have reported that circulating levels of IL-18 are high in patients with both forms of HLH and its levels correlate with the various HLH markers (Takada et al., 1999; Dinarello and Kaplanski, 2005; Mazodier et al., 2005). In addition, we have reported an imbalance of IL-18BP/IL-18 in S-HLH patients leading to an excess of free IL-18, which likely contributes to the large excess of IFN-γ and TNF-α found in these patients (Mazodier et al., 2005). Recombinant human IL-18BP inhibits IL-18 with a Kd in the nanomolar range (Novick et al., 1999), an affinity far greater than the naturally occurring cell receptor. In addition, IL-18BP neutralizes IL-18 at concentrations significantly lower than soluble forms (extracellular domain) of the IL-18 receptor alpha chain (Reznikov et al., 2002). Recombinant human IL-18BP has been shown to be as potent as the natural IL-18BP and to be safe in preliminary human trials conducted in psoriasis or rheumatoid arthritis (Dinarello and Kaplanski, 2005; Tak et al., 2006; Dinarello, 2007). We took advantage of the absence of species specificity of IL-18BP and tested it in a model of MCMV-infected Perf1 KO mice to determine whether IL-18 could be a target for the treatment of HLH in humans.

## Results

### Perforin-deficient mice infected with MCMV: An animal model of HLH

**Figure 1:**
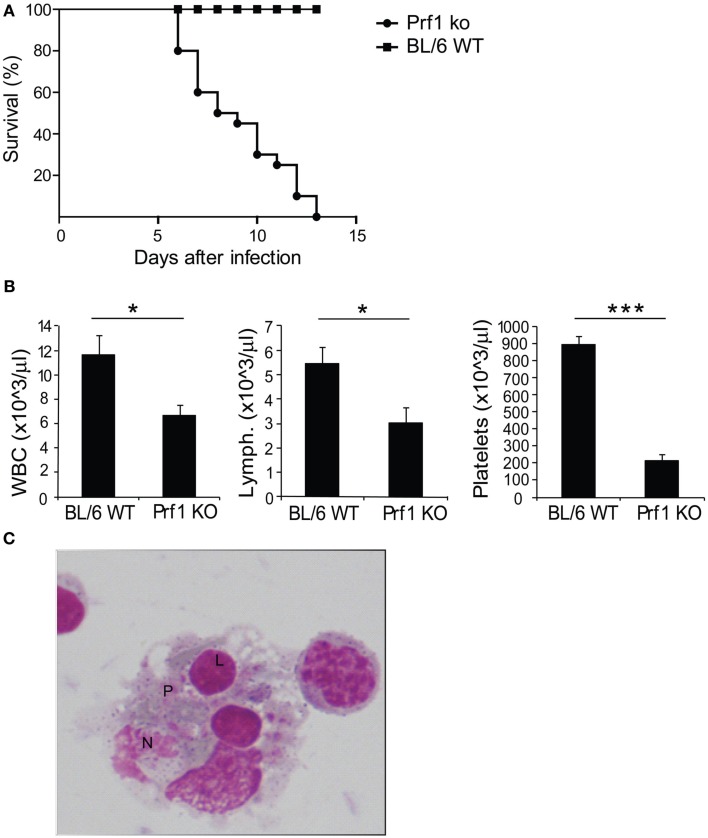
**Prf1 KO mice infected with mouse CMV (MCMV) display increased mortality compared to BL/6 WT mice and develop an HLH-like phenotype**. **(A)** Kaplan–Meier survival curves: BL/6 WT mice and Prf1 KO mice were infected with 1.5 × 10^4^ PFU of MCMV WT and survival was monitored for 15 days after infection. *N* = 20 mice per group. **(B)** White blood cell, lymphocyte, and platelet counts in peripheral blood of MCMV-infected BL/6 WT and Prf1 KO mice, 6 days after infection. Cell numbers are expressed as mean ± SEM. *N* = 4 mice per group. *p < 0.05; ***p < 0.001. **(C)** Hemophagocytosis in the bone marrow of MCMV-infected Prf1 KO mice: activated macrophages engulfing platelets (P), lymphocytes (L), neutrophils (N). Magnification ×100.

We examined perforin-deficient mice infected with MCMV to determine whether they could represent a model of HLH. These mice do not spontaneously develop any syndrome, but they are unable to control MCMV infection (Orange and Biron, 1996). When inoculated with 1.5 × 10^4^ PFU of MCMV, BL/6 WT mice normally survived infection, showing no sign of illness, however Prf1 KO mice died between day 6 and day 13 after infection (Figure 1A). Starting from day 3, mice developed severe illness, manifested by progressive cachexia, weight loss, and splenomegaly (data not shown). At day 6 after infection, Prf1 KO mice were pancytopenic affecting leukocytes, lymphocytes, and platelets (Figure 1B). Moreover, the analysis of the bone marrow revealed the presence of activated macrophages with evidence of hemophagocytosis affecting platelets (P), lymphocytes (L), and neutrophils (N; Figure 1C). Thus, Perf1 KO mice infected with MCMV develop a clinical and hematological picture similar to that observed in humans with HLH.

### Blocking IL-18 abrogates histological lesions in MCMV-associated HLH

**Figure 2:**
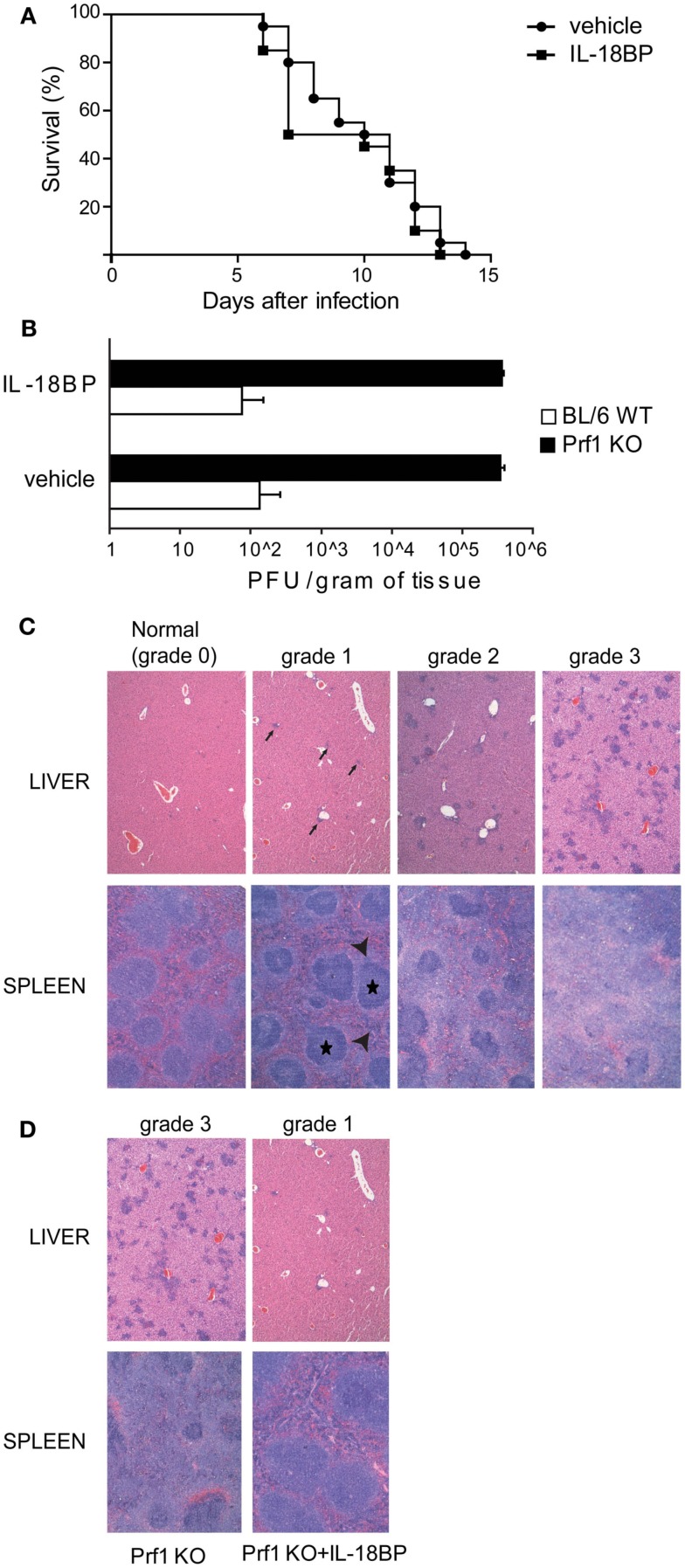
**IL-18BP does not improve Prf1 KO mice survival after mouse CMV (MCMV) infection but consistently reduces inflammatory lesions in the liver and spleen**. **(A)** Kaplan–Meier survival curves for Prf1 KO mice infected with 1.5 × 10^4^ PFU of MCMV, treated or not with IL-18BP. *N* = 20 mice per group. **(B)** MCMV titers in liver homogenates of BL/6 WT and Prf1 KO mice, with or without IL-18BP, 6 days after infection. The data are expressed as mean ± SEM. *N* = 3 mice per group. **(C)** Histological score of liver and spleen sections from MCMV‘infected mice, stained with hematoxylin and eosin. *In the liver*, *grade 1* lesions were defined as near-normal histology with only very few small clusters of inflammatory cells (black arrows); *grade 2* lesions were defined as moderate number of small and non-confluent clusters of inflammatory cells; *grade 3* lesions were defined as numerous, large, and often confluent clusters of inflammatory cells replacing normal hepatic lobules and portal tracts. *In the spleen*, *grade 1* lesions showed only a slight accumulation in the red pulp of mononuclear clear cells (arrow heads) around well preserved islets of white pulp (black stars); *grade 2* lesions were defined as a more prominent accumulation of mononuclear clear cells around less preserved areas of white pulp; *grade 3* lesions were defined as large amounts of mononuclear clear cells accumulating in the red pulp around small remnants of white pulp. **(D)** Histological lesions in MCMV-infected Prf1 KO mice: IL-18BP prevents severe histological damage. Histological score is indicated in the figure. The photomicrographs are representative of three independent experiments each with three mice per group.

We blocked IL-18 using IL-18BP administration in order to test whether this treatment can improve the illness. Prf1 KO and BL/6 WT control mice were infected with MCMV and treated with 10 μg of IL-18BP or vehicle starting 84 h after infection. We chose to block IL-18 at day 3.5 after infection to avoid affecting endogenous antiviral effects, such as the induction of IFN-γ production that contributes to the early control of MCMV infection (Orange and Biron, 1996). IL-18BP administration did not show any effect on mice survival, neither for Prf1 KO mice (Figure 2A) nor for BL/6 WT mice, whose normal resistance to MCMV infection was not altered (not shown). Prf1 KO mice succumbed to MCMV infection with high viral replication; indeed, plaque assay of liver homogenates showed viral titers >1000-fold higher in Prf1 KO mice than in WT mice at day 6 after inoculation (Figure 2B), a time when Prf1 KO mice were in the exacerbation phase of the disease and before they began to die. IL-18BP thus did not affect viral replication in Prf1 KO mice nor in BL/6 WT mice (Figure 2B).

In order to assess the extent of spleen and liver damage in BL/6 WT and Prf1 KO mice after MCMV infection and the possible effect of IL-18BP, we defined a semi-quantitative histological score ranging from normal histology (grade 0) to severe lesions (grade 3; Figure 2C). Six days after MCMV infection, Prf1 KO mice were affected by multiple histologic changes consistent with hemophagocytic syndrome (Figure 2D). Liver and spleen of Prf1 KO mice were seriously damaged by MCMV infection, showing grade 3 lesions whereas after IL-18BP treatment, infected Prf1 KO mice showed grade 1 lesions both in the liver and the spleen (Figure 2D). BL6/WT and BL6/WT treated with IL-18BP all showed normal histology (data not shown). Importantly, hemophagocytosis in bone marrow was also abrogated by IL-18BP treatment. Together, these data indicated that IL-18 is a non-redundant mediator of organ damage, since treatment with IL-18BP abrogated histological lesions induced by MCMV in spleen and liver.

### Treatment with IL-18BP reduces IFN-γ and TNF-α production

**Figure 3:**
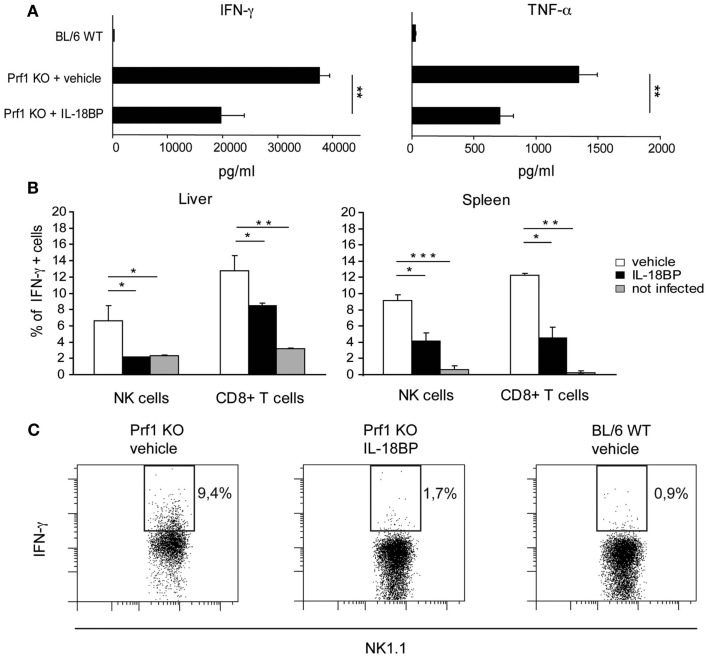
**IFN-γ and TNF-α production is reduced upon treatment with IL-18BP**. **(A)** Serum concentrations of IFN-γ and TNF-α in BL/6 WT and Prf1 KO mice 6 days after mouse CMV (MCMV) infection were determined by ELISA. Mean ± SEM of three independent experiments, *N* = 5 mice per group. **p < 0.01. **(B)** Frequency of IFN-γ^+^ NK cells and CD8^+^ T lymphocytes from liver and spleen of Prf1 KO mice 6 days after MCMV infection. Results are expressed as mean ± SEM of four mice per group. *p < 0.05; **, *p* < 0.01; ***p < 0.001. **(C)** Representative flow cytometric analysis of IFN-γ expression by liver NK cells from vehicle treated Prf1 KO mouse, IL-18BP treated Prf1 KO mouse, BL/6 WT mouse, 6 days after MCMV infection.

In HLH, organ damage is triggered by high levels of proinflammatory cytokines, which activate histiocytes and promote the infiltration of inflammatory cells. Notably, in Prf1 KO mice developing HLH following MCMV infection, TNF-α and IFN-γ contribute to the pathogenesis of the disorder, as specific blockade of either cytokine is protective (Orange and Biron, 1996; Van Dommelen et al., 2006). Since IL-18 induces cytokines and chemokines, up-regulates VCAM–1 in hepatic sinusoidal endothelial cells as well as stimulates the production of IFN-γ and TNF-α (Dinarello, 2007), we tested whether IL-18BP administration could reduce the levels of these cytokines. Serum concentrations of IFN-γ and TNF-α were measured in Prf1 KO and BL/6 WT mice 6 days after MCMV infection (Figure 3A). The level of both cytokines was greatly increased in Prf1 KO mice compared to BL/6 WT mice, but treatment with IL-18BP significantly reduced by half the serum levels of both IFN-γ and TNF-α (Figure 3A). In order to assess the source of IFN-γ, we measured the levels of cytoplasmic IFN-γ by flow cytometry in liver and spleen cell suspensions from Prf1 KO mice 6 days after MCMV infection, without any further *in vitro* stimulation. Both NK cells and CD8^+^ T lymphocytes contributed to IFN-γ production during the late phase of MCMV infection (Figure 3B). IL-18BP reduced IFN-γ production in CD8^+^ T cells as well as in NK cells in the spleen. Interestingly in the liver, IL-18BP suppressed IFN-γ production by NK cells to a greater degree than in T cells, reducing levels to those observed in uninfected mice (Figures 3B,C).

### Antiviral drug and IL-18BP combination therapy blocks the mediators of organ damage

**Figure 4:**
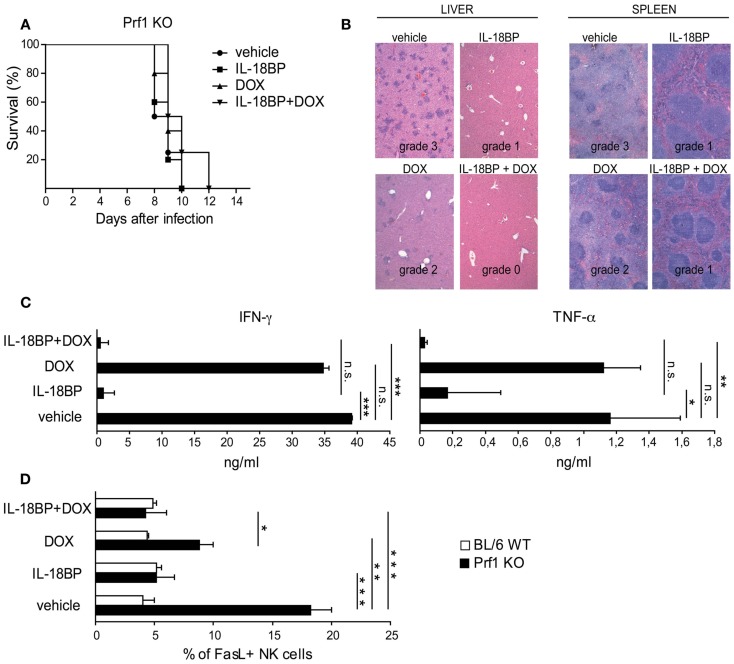
**IL-18BP combined with doxycycline (DOX) abrogates histological lesions and reduces the production of the mediators of organ damage**. **(A)** Kaplan–Meier survival curves: Prf1 KO mice were infected with DN-SCP-MCMV and treated with IL-18BP, doxycycline, or both. Survival was monitored for 15 days post-infection. *N* = 5 mice per group. **(B)** Histological analysis of liver and spleen from Prf1 KO mice infected with DN-SCP-MCMV: hematoxylin and eosin stained sections, representative of three mice per group. Histological score is indicated in the figure. **(C)** IFN-γ and TNF-α serum concentrations from Prf1 KO mice 6 days after infection with DN-SCP-MCMV and treated with IL-18BP, DOX, or both. Mean ± SEM of three mice per group. *p < 0.05; **p < 0.01; ***p < 0.001; n.s., not significant. **(D)** Fas ligand (FasL) expression in liver NK cells derived from Prf1 KO mice and BL/6 WT mice 6 days after DN-SCP-MCMV infection and treated with IL-18BP, doxycycline, or both. Mean ± SEM frequency of FasL positive NK cells in Prf1 KO mice and BL/6 WT mice. *N* = 3 mice per group. *p < 0.05; **p < 0.01; ***p < 0.001.

**Figure A1:**
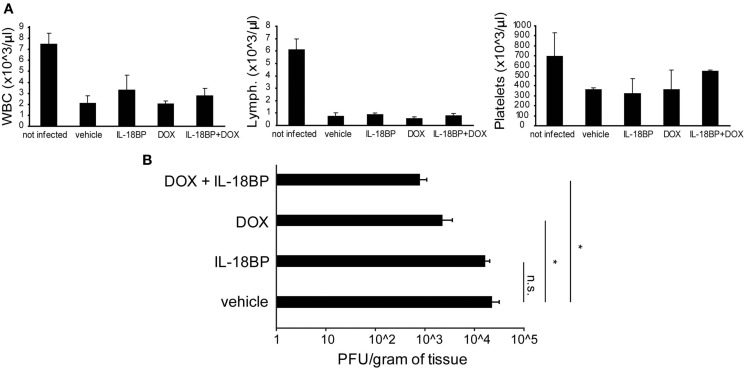
**Doxycycline and IL-18BP combination therapy**. Prf1 KO mice were infected with DN-SCP-MCMV and treated with IL-18BP, DOX, or both. Six days after infection mice were sacrificed and different parameters were evaluated. **(A)** White blood cell, lymphocyte, and platelet counts in peripheral blood, expressed as the mean ± SEM of four mice per group. *p < 0.05; n.s., not significant. **(B)** Viral titers in the liver, expressed as the mean ± SEM of three mice per group.

In Prf1 KO mice, HLH is triggered by MCMV infection but as viral replication increases rapidly, the infection becomes lethal. In an attempt to mimic the treatment option in humans by combining anti-infectious drugs with an immunomodulatory agent, we used a genetically engineered MCMV strain, DN-SCP-MCMV, for which *in vivo* replication can be arrested by the administration of doxycycline (DOX; Robbins et al., 2007). Prf1 KO mice were infected with 1 × 10^6^ PFU of *in vitro*-derived DN-SCP-MCMV and starting 84 h after infection, mice received DOX, IL-18BP, or a combination of both. All Prf1 KO mice infected with DN-SCP-MCMV developed a severe illness with profound leukopenia and thrombocytopenia (Figure A1A in Appendix). As shown in Figure 4A, treatment with DOX was not sufficient to rescue Prf1 KO mice, as mice succumbed by day 10. Consistent with this observation, viral burdens in liver homogenates were determined 6 days after infection by plaque assay and we unfortunately observed that DOX was not sufficient to totally block viral replication in our mice (Figure A1B in Appendix). DN-SCP-MCMV-infected mice treated with IL-18BP alone also succumbed by day 10, but although it did not reach statistical significance, a slight trend toward a better survival up to day 12 was nevertheless observed when the mice received a combination of DOX and IL-18BP (Figure 4A).

The histological analysis of the liver and spleen of Prf1 KO mice infected with DN-SCP-MCMV exhibited the same grade 3 lesions than those observed in Prf1 KO mice infected with MCMV (Figure 4B). Consistent with the lack of complete effects of DOX on viral replication, Prf1 KO mice infected with DN-SCP-MCMV and treated with DOX showed only minimal histological improvement (grade 2), whereas a combination of DOX and IL-18BP significantly improved histological damages to grade 1 and grade 0 in the liver and spleen, respectively.

In order to characterize the mechanism of action of IL-18BP in these experimental conditions, we examined changes in IFN-γ, TNF-α, and Fas ligand (FasL). In order to compare with the observations obtained in Prf1KO mice infected with WT MCMV, IFN-γ and TNF-α were quantified by ELISA in the sera of Prf1 KO mice infected with DN-SCP-MCMV. Similar to the WT MCMV, the engineered virus DN-SCP-MCMV also induced high levels of both cytokines (Figure 4C). Treatment with DOX that only partially blocked viral replication (Figure A1B in Appendix), likewise was not effective in the control of IFN-α and TNF-α levels. On the contrary, IL-18BP administration significantly reduced serum concentrations of both cytokines, confirming data observed in Prf1 KO mice infected with WT MCMV. Furthermore, combination of the antiviral effect of DOX with IL-18BP resulted in the abrogation of IFN-γ and TNF-α production (Figure 4C). FasL was measured by flow cytometry on NK cells derived from Prf1KO and BL/6 WT mice livers, 6 days after infection with DN-SCP-MCMV. In infected Prf1 KO mice, FasL expression was increased compared to infected WT mice (Figure 4D) and was consistently reduced by the administration of DOX and more strongly by those of IL-18BP. Thus, despite the lack of efficacy of DOX treatment to eliminate DN-SCP-MCMV infection, these experiments confirm with another viral strain the beneficial role of IL-18BP in treating the inflammatory syndrome observed in Prf1 KO mice infected with MCMV.

## Discussion

Since the levels of free IL-18 are unusually high and correlate with disease severity in humans with HLH (Dinarello and Kaplanski, 2005; Mazodier et al., 2005) the present study was conducted to evaluate the therapeutic potential of neutralizing IL-18 with IL-18BP in a mouse model of HLH. We subjected Perf1 KO mice to infection with MCMV, which induces IFN-γ and TNF-α (Van Dommelen et al., 2006) and observed that these mice indeed developed a lethal condition similar to HLH, with pancytopenia, hemophagocytosis in the bone marrow and severe inflammation in the liver and spleen. We observed that IL-18BP treatment alone decreased hemophagocytosis and dramatically reversed liver and spleen damage induced by MCMV infection consistently with decreased levels of both IFN-γ and TNF-α and reduced expression of FasL on NK cells. However, and different from LCMV-infected Perf1 KO mice treated with anti-IFN-γ (Jordan et al., 2004), survival was not improved in MCMV-infected Perf1 KO mice treated with IL-18BP. This was not due to a viral burden increase due to IL-18 and it seems unlikely that higher doses of IL-18BP would have reduced lethality, since IL-18BP at the present dose was very effective in reducing both IFN-γ and TNF-α. In this model, complete control of MCMV replication was not achieved despite the use of a genetically designed antiviral strategy with DOX. Since immune defenses against MCMV infection depend on perforin-mediated mechanisms (Bukowski et al., 1984; Orange and Biron, 1996; Van Dommelen et al., 2006), persistent infection with this cytopathic virus was probably responsible for animal death.

HLH appears to result from an infectious trigger in patients with compromised lymphocyte cytotoxicity, either due to genetic mutations in F-HLH or to poorly understood conditions in S-HLH (Grom, 2004; Mazodier et al., 2005; Henter et al., 2007; Pachlopnik Schmid et al., 2010). The common paradigm concerning HLH pathogenesis involves a two-step mechanism: first, polyclonal CD8^+^ T cell proliferation takes place due to continuous infectious stimulation leading to large excess IFN-γ production. High levels of IFN-γ induce in a second step, macrophage activation, with production of inflammatory cytokines and hemophagocytosis (Jordan et al., 2004; Pachlopnik Schmid et al., 2010). This concept is largely the result of studies performed in LCMV-infected Perf1 KO mice, the more accepted animal model of HLH to date (Kägi et al., 1994; Walsh et al., 1994; Jordan et al., 2004). Indeed it appears from this model that uncontrolled proliferation of non-cytotoxic CD8^+^ T cells is the central event leading to excess IFN-γ production and HLH (Kägi et al., 1994; Walsh et al., 1994; Jordan et al., 2004). However, LCMV by itself induces profound CD8^+^ T cell proliferation and activation, even in wild-type animals and immune defenses against this virus have been shown to rely on IFN-γ produced by CD8^+^ T cells as well as perforin-mediated CD8^+^ T cell cytotoxicity, independently of HLH occurrence (Kägi et al., 1994; Badovinac et al., 2003). In LCMV-infected Perf1 KO mice, NK cells do not appear to be involved in HLH pathogenesis, but immune defenses against LCMV do not involve NK cells (Bukowski et al., 1983). In addition, in LCMV-infected Perf1 KO mice, blocking IFN-γ improves survival, whereas blockade of either TNF-α, IL-2, IL-12, or IL-18 was inefficient (Jordan et al., 2004). This may appear quite surprising since IL-12 and IL-18 are potent IFN-γ inducers *in vivo* and are indeed found in large concentrations in these mice (Jordan et al., 2004). Although clinically inconsistent, other therapies have reported the beneficial effects of cyclosporin, anti-TNF-α blocking agents, or anti-CD25 mAb in human HLH (Prahalad et al., 2001; Henter et al., 2007; Olin et al., 2008). Although conclusions drawn from LCMV-infected Perf1 KO mice may be relevant to human HLH induced by non-cytopathic virus such as EBV, it is also a possibility that these conclusions would be different if infection with another virus was used.

In humans, S-HLH can complicate more severe situations such as sepsis or infections due to cytopathic virus, notably CMV or H5N1 and H1N1 influenza virus (Yuen et al., 1998; Riedemann et al., 2003; Rouphael et al., 2007; To et al., 2010). The MCMV-infected Perf1 KO model used in this study may be more relevant to such situations, since MCMV is a cytopathic virus (Bukowski et al., 1983, 1984; Van Dommelen et al., 2006). NK cell perforin-mediated cytotoxicity is essential for the control of MCMV infection in the liver, thus in Perf1 KO mice, MCMV persists in tissues, notably the liver, inducing severe tissue damage due to continuous T and NK cell activation (Van Dommelen et al., 2006). Both IFN-γ and TNF-α have been shown to be involved in tissue damage and anti-TNF-α treatment is partly protective (Van Dommelen et al., 2006). Liver involvement is consistently observed in human HLH; CD8^+^ T cells and macrophages/histiocytes are frequently observed to infiltrate the liver and it is liver failure that signals a severe prognosis of the disease (Chen et al., 2010). In other disease models involving the liver, IL-18 plays an important role through different mechanisms (Tsutsui et al., 2000). For example, IL-18 acts in synergy with IL-12 for IFN-γ production by hepatic T lymphocytes which in turn induces TNF-α secretion by Kupffer cells (Tsutsui et al., 2000). IL-18 by itself or in synergy with IL-2 is also a potent inducer of NK cell cytotoxicity through activation of the perforin and FasL pathways (Dao et al., 1998; Tsutsui et al., 2000). In IL-18 transgenic mice, hepatic lesions are entirely mediated by NK and FasL/Fas mechanisms and hepatocytes spontaneously express Fas (Finotto et al., 2004). Similarly, in various murine models, IL-18 in combination with IL-12 or IL-2 exerts its anti-tumoral effects through activation of NK cells and FasL pathways (Osaki et al., 1998; Wigginton et al., 2002). In the present study, we observed that both liver-infiltrating CD8^+^ T cells and NK cells are indeed strong producers of IFN-γ and TNF-α and that these two cytokines could be largely reduced by IL-18BP treatment. In addition, we observed that expression of FasL was markedly increased on NK cells in the liver but nearly completely prevented by IL-18BP treatment. From these studies the concept emerges that FasL is likely causative in HLH tissue lesions, in agreement with previous reports showing that high levels of soluble FasL circulate in humans with HLH (Hasegawa et al., 1998). Since IFN-γ induces Fas expression on cell membranes (Tsutsui et al., 2000), inhibition of IFN-γ production by IL-18BP can also decreased Fas expression on target cells and IL-18BP may thus completely inhibit this cell death mechanism. Moreover, the ability of FasL/Fas to induce IL-18 takes place independent of caspase-1 (Tsutsui et al., 1999). Thus, beside its protective property reducing liver or spleen cell death, inhibition of FasL-Fas interactions by IL-18BP may defeat an amplification loop of IL-18 secretion, which can be locally deleterious.

S-HLH treatment in humans relies on different strategies such as anti-infectious agents combined with steroids or cyclosporin (Ravelli, 2002; Henter et al., 2007; Janka, 2007). In refractory cases, etoposide is frequently used and in F-HLH, the International Histiocyte Society guidelines recommend the combined use of steroids, etoposide, cyclosporin and intrathecal methotrexate before bone marrow transplantation in case of recurrence (Henter et al., 2007). This treatment regimen, although effective, is not without serious side effects and a case of acute leukemia occurring in a patient previously treated for EBV-associated HLH has been recently reported (Sathiyamoorthy et al., 2011). Because of its possible side effects, etoposide is not commonly used in patients with S-HLH, for example, in patients with auto-immune or inflammatory diseases. There is clearly a need for new treatments. In this context, anti-TNF-α agents, anti-CD25, or IL-1 receptor antagonist have been used with varying degrees of success (Prahalad et al., 2001; Behrens et al., 2006; Olin et al., 2008). In view of the data obtained in animal models of HLH using LCMV infection, identifying IFN-γ as a major pathogenic player in HLH pathogenesis, it has been suggested to use an anti-IFN-γ therapy (Pachlopnik Schmid et al., 2009, 2010).

Targeting IL-18 with IL-18BP is thus a rationale mechanism to reduce IFN-γ in HLH since we have observed that IL-18BP concentrations are inadequately elevated in S-HLH patients in comparison with increased IL-18, leading to large excess of free IL-18 which induces IFN-γ (Dinarello and Kaplanski, 2005; Mazodier et al., 2005). The IL-18BP promoter contains an IFN-γ-responsive element and IFN-γ is an effective inducer of IL-18BP production (Paulukat et al., 2001; Hurgin et al., 2002). A failure in the ability of IFN-γ to induce IL-18BP may explain the IL-18/IL-18BP imbalance observed in HLH. In agreement with this hypothesis, a report has shown that in a FHL-type 2 patient, *in vitro* IFN-γ-induction of IL-18BP was markedly reduced (Nold-Petry et al., 2010). These observations in humans together with the benefit of IL-18BP protection against tissue damage in an animal model of HLH may support the use of IL-18BP in humans afflicted with this disease. Therefore, the use of effective antiviral drugs to decrease viral replication combined with IL-18BP to control organ damage induced by the overwhelming inflammatory reaction may be a particularly attractive therapeutic option for humans with HLH.

## Material and Methods

### Mice, viruses, and *in vivo* treatments

C57BL/6 mice were purchased from Charles River Laboratories (L’Arbesle, France). Prf1 KO mice on C57BL/6 background were obtained from Jackson Laboratory (Bar Harbor, ME). All mice were used between 6 and 14 weeks of age. All experiments have been performed in accordance with protocols approved by the local ethical committee. MCMV infections were established on day 0 by i.p. injection of 1.5 × 10^4^ PFU of salivary gland propagated MCMV of Smith strain WT (Henter et al., 2007) or 1 × 10^6^ PFU of *in vitro*-derived DN-SCP-MCMV (Janka, 2007). These modest doses were chosen because they do not induce mortality in BL/6 WT mice. IL-18BP treatment consisted of daily i.p. injections of 10 μg per mouse of recombinant human IL-18BP (a generous gift from Merck Serono, Geneva, Switzerland), starting 84 h after infection. Vehicle was saline. For the *in vivo* arrest of DN-SCP-MCMV replication, 200 μg DOX (Sigma) was delivered by i.p. injection 84 h after infection followed by the addition of 2 mg/ml DOX plus 5% sucrose in drinking water. In survival experiments, mice were infected and assessed for mortality at least once daily for 15 days.

### Serum cytokines detection and complete blood counts

Six days after MCMV infection mice were bled from the retro-orbital plexus and the concentrations of IFN-γ and TNF-α in the serum were assayed by ELISA (R&D Systems, Minneapolis, MN, USA). For complete blood cell counts, blood samples were run a blood cell counter ABX Pentra 60 C+ Horiba Medical (Montpellier, France).

### Histology and analysis of hemophagocytosis

Six days after MCMV infection, mice were sacrificed and the spleens and livers removed. Organs were fixed in 10% buffered formalin in saline, embedded in paraffin, sectioned, stained with hematoxylin, and counterstained with eosin. Cell suspensions were prepared from bone marrow and analyzed for the presence of hemophagocytosis after staining with May-Grünwald-Giemsa.

### Viral titer quantification

Six days after MCMV infection mice were sacrificed and livers were collected, weighted, and homogenized. Viral titers were determined by a standard plaque assay using NIH-3T3 cells.

### Flow cytometry and intracellular IFN-γ quantification

Lymphocyte suspensions were prepared from livers and spleens as described previously (Walzer et al., 2007) and then incubated for 4 h in the presence of GolgiStop (BD Biosciences, San Jose, USA). Cells were then harvested and incubated with the following monoclonal antibodies against cell surface antigens: PerCP anti-NK1.1, APC anti-mouse TCRβ, Pacific Blue anti-mouse CD4 and APC-H7 anti-mouse CD8 from BD Pharmingen (San Diego, CA, USA); PE anti-mouse CD178 (Fas Ligand) from e-Bioscience (San Diego, CA, USA). For intracellular staining, samples were treated with BD Cytofix/Cytoperm and Perm/Wash solutions (BD Biosciences). IFN-γ production was detected by PE-Cy7 anti-mouse IFN-γ antibody (BD Pharmingen). Samples were run on a FACS Canto II (Becton-Dickinson, San Jose, USA) and analyzed using Flow Jo 8.7 software (TreeStar Inc., Ashland, OR, USA) and Sw Gatelogic (IniVai Technologies, Mentone Victoria 3194, Australia).

### Statistical analyses

Statistical analyses were performed via GraphPad Prism, using the unpaired two-tailed Student’s *t*-test. n.s.: not significant; *p < 0.05; **p < 0.01; ***p < 0.001.

## Conflict of Interest Statement

Eric Vivier is a cofounder and shareholder of Innate-Pharma.
